# The Influence of Vinification Methods and Cultivars on the Volatile and Phenolic Profiles of Fermented Alcoholic Beverages from Cranberry

**DOI:** 10.3390/antiox8050144

**Published:** 2019-05-23

**Authors:** Jingying Zhang, Donglin Chen, Xiao Chen, Paul Kilmartin, Siew Young Quek

**Affiliations:** 1 Food Science, School of Chemical Sciences, The University of Auckland, Auckland 1010, New Zealand; zjy0710@gmail.com (J.Z.); tony.chen@auckland.ac.nz (D.C.); xche622@aucklanduni.ac.nz (X.C.); p.kilmartin@auckland.ac.nz (P.K.); 2 Riddet Institute, New Zealand Centre of Research Excellence for Food Research, Palmerston North 4474, New Zealand

**Keywords:** cranberry, wine, fermentation, phenolic, volatile, LC-MS/MS, GC-MS, PCA

## Abstract

This study investigated the effects of vinification techniques and cultivars (Stevens, Pilgrim and Bergman) on cranberry wine quality. Three winemaking technologies were conducted to prepare cranberry musts before fermentation, including traditional red and white vinifications (Red and White), and thermovinification (Thermo). In wine products, proanthocyanins (PACs) and anthocyanins (ANCs) are dominant in phenolics, while esters and alcohols are prevalent in volatiles, with phenylethyl alcohol, β-damascenone, benzyl alcohol, etc. as the main contributors to the aroma. The phenolic compositions of wines were in the same pattern with cultivars: the Stevens and Bergman wines contained the highest amount of ANCs and PACs, respectively, while the Pilgrim wines had the lowest total phenolic contents (TPC), and antioxidant capacities (AOC). Nevertheless, products from Pilgrim cultivar had a distinctive pattern of volatiles compared to Stevens and Bergman, especially for aromatic compounds. Considering vinification methods, Thermo demonstrated advantages on correlations with both phenolic and volatile (polymeric and monomeric) compositions.

## 1. Introduction

American cranberries (*Vaccinium macrocarpon* Ait.) and related products have attracted high public interest over the past twenty years. Cranberry contains abundant Vitamin C and polyphenols (proanthocyanins, anthocyanins, flavonols, and phenolic acids), which are responsible for various health benefits (antioxidant, anti-bacterial, antitumor, anti-inflammatory activities) [1,2,3], making cranberry a promising source of beneficial phytochemicals. Cranberry consumption has been correlated with women’s health, based on the protective effects of cranberry on urinary tract infection [4]. Different from other berries, cranberries are rarely eaten raw, due to the low acceptance of the fresh fruit flavour [5]. In contrast to blueberries (12.74% total sugar) [6], fresh cranberries do not taste sweet (only 4% total sugar), but are rather sour (pH < 3.0) and bitter (due to high tannins content) [5]. Therefore, cranberries are mainly consumed as processed products. Cranberry juice (takes up 60%), jam, syrup, and sundried fruits are the most common processed cranberries, and all of these products are sweetened [5,7]. At the same time, cranberries have also been included in some yoghurt products, functional powders, and beverages [8].

Fermentation is the process of converting sugars into alcohols, esters, and other volatiles, through the action of yeasts. Production of alcoholic beverages from fruits other than grapes has been a new trend in recent years. Various fruits, including pomegranate, berries, apple, mango, banana, lychee, peach, kiwi, and cherry have been used for fermented beverages [9,10]. Though cranberry juice has been used as a raw ingredient in some commercial alcoholic beverages, there are few pieces of literature reporting cranberry wine characterization. Rupasinghe and Clegg compared total antioxidant capacity, total phenolic content, mineral elements, and histamine of 14 different commercial wines, including cranberry wine, and found cranberry wine with the highest content of calcium [11]. Vuong, et al. attempted to ferment cranberry wine utilizing a novel bacteria, and obtained satisfactory wine product with high antioxidant activity [12]. Nevertheless, research about cranberry fermentation is still insufficient, and the gaps such as vinification methods, phenolic profiles and aroma, need to be filled. Most of the current research about cranberry focused on its phytochemicals and health benefits. Cranberry has been proven to be a better source of flavonoids than grapes, and the protective potentials of cranberry are well studied now [13]. Winemaking can be considered as a promising option for cranberry processing, which can potentially retain cranberry’s aroma and the health beneficial compounds, as well as bioactivities while producing a characteristic product for good consumer acceptability.

Considering functional food or beverage products, consumers are most interested in the health-promoting features and antioxidant activities of the products. At the same time, the volatile composition will define the specificity or uniqueness of aroma present in alcoholic beverage, playing a key role in distinguishing the relevant products with regard to their quality, and consumer acceptability and preferences. Consequently, the quality of cranberry alcoholic beverage will be closely correlated with phenolic and volatile profiles, which are dependent on the fruit origin, cultivar, yeast strains, and vinification procedures.

Cranberry is a relatively novel crop in New Zealand (commercially growth for around 20 years) in comparison with the long cultivation history in North America [14]. However, there are few scientific reports about New Zealand grown cranberries, not to mention processing them into products. This is the first report on the production of novel fruit wine by fermentation, utilizing New Zealand grown cranberries. Three cultivars (Stevens, Pilgrim and Bergman) were studied in this work. Given the diversity of phytochemicals found in various cultivars, differences in the final wine characteristics are expected, and this warrant the current investigation.

Traditional white vinification (white wines) utilizes the clear juice after removing the skin and seeds [15]. In contrast, traditional red vinification (red wines) utilizes skin maceration without removing the solids from the juice, and the skin contact can increase the polyphenol concentrations apparently by continuous extraction during fermentation [16]. At the same time, a novel technique, pre-fermentation heat treatment or thermovinification is becoming more popular in the grape wine industry, through which the volume of wines produced in France in 2017 was around 750 million litres [17]. Thermovinification can produce fruity red wines with soft tannins, by heating grape must to 70–75 °C for 30 min to 24 h before fermentation [18]. Thermovinification was reported to enhance the phenolic compounds, colours and also the grape-derived aromas [17,19]. The vinification methods were well studied on red and white wine fermentations but were rarely investigated on fruit wine. Considering the different chemical characteristics of cranberry from red or white grapes, it is worthwhile to study the effects of various vinification methods on cranberry wine quality. In this study, three different vinifications were conducted prior to fermentation to prepare three cranberry musts: whole fruit juice without further treatment as traditional red vinification (Red), clear juice obtained by filtering as traditional white vinification (White), and heated juice by thermovinification before filtering (Thermo). The related influence was evaluated by characterization of phenolic and volatile profiles of the final cranberry wines.

The objective of this research was to study the effects of cultivars (Stevens, Pilgrim, and Bergman) and the vinification techniques (Red, White, and Thermo) on the cranberry alcoholic beverage quality, with volatile and phenolic profiles as main parameters. The understanding of the relationship between chemical characteristics of cultivars and final products is significant to provide a reference for other phenol-rich fermentation beverages.

## 2. Materials and Methods

### 2.1. Chemicals and Reagents

Trolox (6-hydroxy-2,5,7,8-tetramethylchroman-2-carboxylic acid), 2,2-azinobis-(3-ethylbenzothiazoline-6-sulfonic acid)-diammonium salt (ABTS), 2,4,6-tripyridyl-s-triazine (TPTZ), p-dimethylaminocinnamaldehyde (DMAC), Folin-Ciocalteu reagent, phenolic standards (catechin, epicatechin, quercetin, gallic acid, vanillic acid, protocatechuic acid, syringic acid, *p*-coumaric acid, ferulic acid, caffeic acid, *trans*-sinapic acid, Phloridzin) were purchased from Sigma-Aldrich (St. Louis, MO, USA). Other high purity analytical phenolic standards (procyanidin B1, B2 and A2, cyanidin 3-galactoside, cyanidin 3-glucoside, cyanidin 3-arabinoside, peonidin 3-glucoside, myricetin-3-galactoside, myricetin-3-glucoside, quercetin-3-galactoside, and quercetin-3-rhamnoside) were from Extrasynthese (Genay, France). HPLC grade chemicals, e.g., formic acid, were from BDH Chemical Ltd., Co. (Poole, England) and acetonitrile from Romil Ltd., Co. (Cambridge, England). Potassium metabisulfite (PMS) was from Enartis (Trecate, Italy). All other chemicals used were of analytical grade. Type 1 water (Barnstead NANOpure^®^ Diamond™, Thermo Scientific, Waltham, MA, USA) was used in experiments.

For GC-MS, deuterated internal standards of high purity, including d_3_-ethyl butyrate, d_11_-ethyl hexanoate, d_15_-ethyl octanoate, d_3_-3-methylbutyl acetate, d_3_-n-hexyl acetate, d_3_-2-phenylethyl acetate, d_3_-linalool, d_3_-α-Terpineol, d_2_-3-methyl-1-butyl alcohol, d_11_-n-hexyl-2,2,3,3,4,4,5,5,6,6-alcohol, d_5_-2-phenyl alcohol, d_12_-n-hexanal, d_11_-hexanoic acid were from CDN Isotopes (Pointe-Claire, Canada). High purity non-deuterated internal standards: DL-3-octanol was from Acros Organics (Geel, Belgium), 4-decanol from Lancaster (Morecambe, England), and 3-4, dimethylphenol from Aldrich (Castle Hill, Australia). Analytical volatile standards ethyl acetate, pentanal, methyl butyrate, ethyl methylbutyrate, ethyl 2-methylbutanoate, methyl hexanoate, 3-octanone, D-Limonene, eucalyptol, methyl benzoate, methylbutyric acid, ethyl octanoate, ethyl benzoate, β-phenylethyl acetate, β-damascenone, 4-ethylguaiacol, ethyl cinnamate (*trans*), benzoic acid, and β-damascenone were purchased from Aldrich (Castle Hill, Australia). Other volatile standards were purchased from different suppliers as follow: benzyl alcohol from Riedel-de Haën (Lotte, Germany); isobutanol from Scharlau (Barcelona, Spain); isoamyl alcohol from Panreac (Barcelona, Spain); isoamyl acetate from Univar (Downers Grove, IL, USA); 4-ethylphenol from Lancaster (Morecambe, England); phenylethyl alcohol, ethyl butyrate, octanoic acid, decanoic acid, α-terpineol, geraniol (trans), from Acros Organics (Geel, Belgium); ethyl hexanoate, ethyl decanoate, isobutyl acetate, hexanoic acid, from Fluka (Castle Hill, Australia).

### 2.2. Preparation of Cranberry Wine

Three cultivars of fresh cranberries (Stevens, Pilgrim and Bergman) were obtained from Wild Ruby Cranberries, Greymouth, South Island, New Zealand. All fruits were harvested at the fully ripening stage, with a dark-red colour. Cranberries were stored immediately at −18 °C once received until further experiments.

The fermentation process was modified from Yuan [20] and is demonstrated in Figure S1. Fruits were defrosted, washed and blended (1:1 *w*/*w*) with water using a Vitamix Total Nutrition blender (Ohio, USA). The total soluble solid content of the juice was determined using the Atago Hand-Held refractometer (model ATC-IE, Brix 0–32%, USA). The commercial enzyme of Lallzyme EX-V (Lallemand, France) was added to the must at 400 mg/L, which was then incubated at 40 °C for 3 h for enzyme hydroxylation. The must was divided into three groups according to treatments before fermentation, namely: Red (whole fruit juice), White (pomace removed by filtering), Thermo (incubated at 70 °C for 3 h and pomace removed by filtering). Sucrose was then added to the must to adjust the Brix to 9°. The musts were inoculated by the commercial *Saccharomyces cerevisiae* strain DV 10 (Lallemand, France). Around 700 mL of must was transferred to 750 mL green glass bottles, to which a rubber bung was added and 200 µL plastic pipette tip filled with cotton inserted for CO_2_ released during fermentation. The fermentation was carried out in triplicate at 16 °C, monitored by weighing the bottles daily. Fermentation was considered complete when the weight loss became stable. SO_2_ (20 ppm) was then added to the finished wines (in potassium metabisulphite), which were stored at –4 °C for 2 weeks for cold settling and sedimentation. The final samples were filtered and stored at −80 °C prior to testing. The final samples were labelled as Stevens White (SW), Stevens Thermo (ST), Stevens Red (SR), Pilgrim White (PW), Pilgrim Thermo (PT), Pilgrim Red (PR), Bergman White (BW), Bergman Thermo (BT) and Bergman Red (BR).

### 2.3. Determination of Total Phenolic Content (TPC), Proanthocyanin (PAC) and Monomeric Anthocyanin (ANC) Contents

The TPC of the juices and wines were determined using Folin-Ciocalteau assay, modified from the procedure of Tang et al. (2015), with absorbance measured at 765 nm [21]. The final results were expressed as µg gallic acid equivalent (GAE) per L of wine. The PAC contents of samples were estimated by a modified 4-dimethylaminocinnamaldehyde (DMAC) assay according to Grace et al. at 640 nm [7]. The results were expressed as µg procyanidin A2 (PAC A2) equivalent per L of wine. The ANC contents of the samples were determined using the pH differential method, modified from Lee et al. [22]. The results were expressed as µg cyanidin-3-glucoside (Cyn-3-Glu) equivalent per L of wine. All measurements were carried out in triplicate on an EnSpire Multimode Plate Reader 2300 (PerkinElmer, Waltham, USA).

### 2.4. Determination of Antioxidant Capacity (AOC)

Two different assays, the free radical-scavenging activity method (ABTS) and ferric reducing ability of plasma method (FRAP), were carried out to test the AOC, both modified from Ozgen et al. [23]. All measurements were carried out in triplicate, using an EnSpire Multimode Plate Reader 2300 (PerkinElmer, Waltham, MA, USA). Trolox was used as the standard for both assays. The results were expressed as mmol Trolox equivalent (TE) per mL of wine.

### 2.5. Quantification of Individual Anthocyanins

The analysis of anthocyanins was conducted by HPLC-DAD using an Agilent 1290 HPLC system (Agilent Technologies, Santa Clara, CA, USA) equipped with a diode array detector (DAD). The separation was performed on a Poroshell 120 EC-C18 column (2.1 mm × 100 mm; 4 µm; Agilent Technologies, Santa Clara, CA, USA) at 35 °C. The mobile phase consisted of 3% formic acid in water (A), and 90% Acetonitrile, 5% MeOH and 5% Water (B) at a flow rate of 0.35 mL/min. The gradient elution program was set as follow, modified from Vagiri, et al. [24]: 0–1.5 min, 5% B; 1.5–8 min, 5–15% B; 8–9.5 min, 15–20% B; 9.5–11.5 min, 20% B;11.5–14 min, 20–22% B; 14–20 min, 22–28% B; 20–24, 28–30% B; 24–29 min, 30–90% B; 29–32 min, 90% B; 32–35 min, 90–5% B. The injection volume was 3 µL and the wavelength used for anthocyanin detection was 520 nm. Quantification of cyanidin 3-galactoside, cyanidin 3-glucoside (Cyn-3-Glu), cyanidin 3-arabinoside, peonidin 3-glucoside was carried out using standard curves of the analytical standards. The contents of peonidin 3-galactoside and peonidin 3-arabinoside were determined by the sequence of peaks, and the concentrations were expressed as µg Cyn-3-Glu equivalent per mL of wine.

### 2.6. Quantification of Proanthocyanins, Flavonols, and Phenolic Acids

The detection of phenolic compounds (other than ANC) was conducted by LC-MS/MS using an Agilent 6460C triple quadrupole mass spectrometer equipped with infinity 1290 LC system (Agilent Technologies, Santa Clara, CA, USA). The conditions for separation was similar to the HPLC-DAD as mentioned above. The method was modified from Zhao, et al. [25]. Samples were analysed in negative mode with a gas temperature of 250 °C, gas flow rate of 10 L/min, and Vcap 3500 V. Quantifications were carried out by standard curves of each analytical standard.

### 2.7. Quantification of Volatile Compounds

The volatiles were extracted by headspace-solid phase microextraction (HS-SPME) and separated by GC-MS. The method was modified from Jouanneau, et al. [26]. The standards were grouped into three mixtures according to chemical classes and retention time, in order to optimize the separation. The stock solution was prepared for each mixture and then diluted with a base solution to five calibration levels. Wine and juice base solutions were prepared by adding citric acid and ethanol to water, mimicking the wine (pH 3.0, and alcohol content of 4%) conditions.

Each wine sample/standard solution (10 mL) was placed in a 20 mL amber glass screw-capped SPME vial (75.5 × 22.5 mm, Thermo Scientific, Waltham, MA, USA). After adding 20 µL of internal standard mix and 3 g of NaCl, the vials were purged with nitrogen and tightly capped. The samples were placed on a gerstel multipurpose sampler (MPS) tray. Prior to extraction, the samples were incubated at 40 °C for 10 min in a shaker bath. The volatiles were then extracted by headspace-solid phase microextraction (HS-SPME) for 60 min at 45 °C, with a 2 cm, 23-Gauge, 50/30 µm, DVB/CAR/PDMS fibre (Supelco Inc., Bellefonte, PA, USA). The SPME fibre was baked out at 250 °C for 5 min before and after each analysis to avoid possible contamination.

After extraction, the detection was conducted on an Agilent Technologies 7890 gas chromatograph system coupled with a 5975C inert XL MSD (Agilent Technologies, Santa Clara, CA, USA). The volatiles in the extract were separated using a tandem column composed of an HP-1MS column (30 m, 0.320 mm ID, 0.25 µm film), and an HP-INNOWax-fused silica capillary column (30 m, 0.320 mm ID, 0.25 µm film) (Agilent Technologies, Santa Clara, CA, USA). The carrier gas was helium at a flow rate of 1 mL/min. The interface line temperature was 250 °C. The oven conditions were set up as follow: started at 40 °C (5 min), then ramped to 200 °C at the rate of 2 °C/min (80 min), and held for 5 min. The ion source temperature was held at 230 °C, operating in electron impact mode at 70 eV. All samples and standards were run in duplicates. The volatile compounds were identified by authentic standards, and quantified using standard curves of the reference compounds.

### 2.8. Odour Activity Values (OAV)

The OAV of the compounds were calculated by dividing the concentrations by sensory thresholds, according to the literature [27].

### 2.9. Statistical Analysis

Principal component analysis (PCA) was conducted to investigate the relationship between the compounds and wine samples using the Unscrambler X software, version 10.4 (Oslo, Norway). Significant differences (*p* < 0.05) between mean values were evaluated using one-way analysis of variance (ANOVA) Tukey^ab^ test (JMP version 14, Cary, NC, USA). In addition, volatile data were subjected to two-way ANOVA of treatments (cultivars and vinifications) by JMP (Cary, NC, USA).

## 3. Results and Discussion

### 3.1. Chemical Analysis

The initial chemical characteristics of cranberry musts revealed the diversity of the three selected cultivars on the physico-chemical parameters (Table 1). The pH and Brix were not much different among the three cultivars (*p* > 0.05). For AOC, Stevens and Bergman cultivars had similar values, both higher than the Pilgrim cultivar, regardless of the test conducted. Stevens had the highest ANC, while Bergman had the highest TPC and PAC. Among the three cultivars tested, Pilgrim had the least TPC, ANC, PAC and AOC. There have been many reports studying the chemical diversity of different cranberry cultivars, with various results of the phenolic composition of these three cultivars. Some studies reported similar performance of these three cultivars [28,29,30], while other articles draw out opposite conclusions [31]. Even compared with New Zealand grown cranberries (North Island), the results of our study varied on the ANC and PAC contents of these three cultivars [32]. The literature has revealed the significant effects of growing location on the phenolic compositions. A previous study compared the proanthocyanin profiles of different cranberry cultivars from different producing countries, and the results revealed more difference in growing locations than cultivars [33]. This difference in the composition in similar cultivars is possibly associated with geographical locations, and also due to the variations in climate, soil and other environmental factors.

At the end of fermentation, the average ethanol content of the final cranberry wine products was 3.72% ± 0.44%. The alcoholic contents were not much diversified among samples because of the Brix adjustment of the musts before fermentation. The degree of alcohol was low as we pursued, as we aimed to produce low alcoholic functional beverage (3.5% to 4% Alc), targeting females as the main consumers. The pH and titratable acidity (TA) of wines were 3.06 ± 0.06 and 1.34 ± 0.10 g tartaric acid /100 mL, respectively, which are similar to the musts before fermentation.

**Figure 1:**
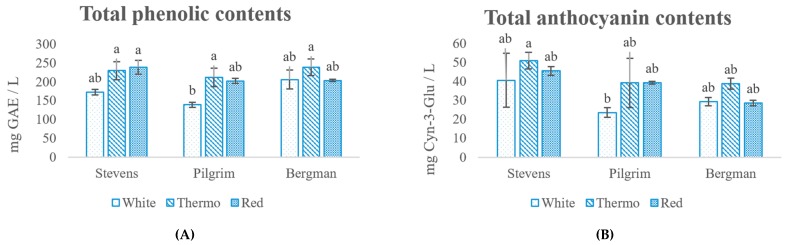

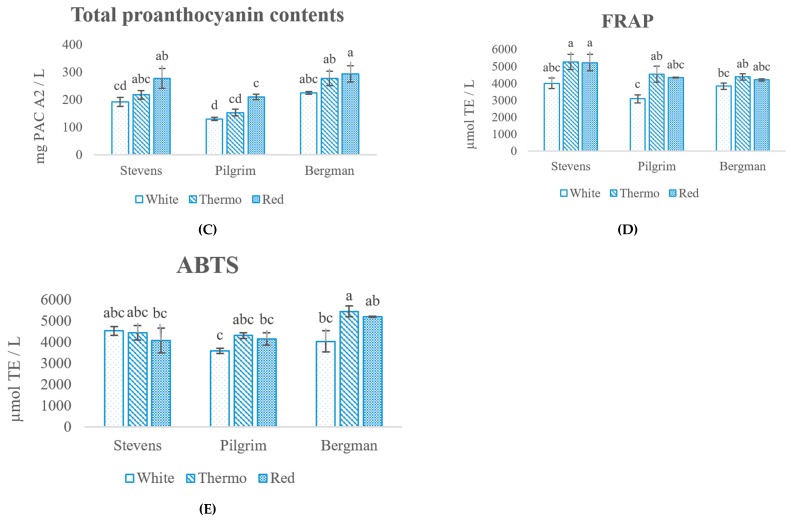
Total phenolic (TPC) (**A**), anthocyanin (ANC) (**B**), proanthocyanin (PAC) (**C**) contents and antioxidant capacities (AOC) (**D**) FRAP (ferric reducing ability of plasma method), (**E**) ABTS (free radical-scavenging activity method) of the wine samples determined by spectrophotometric assays. Values identified by the same letters are not significantly different at the 0.05 level (One-way ANOVA-Tukey^ab^).

The total AOC values of the nine wine samples produced were compared using the FRAP and ABTS assays (Figure 1D). The ABTS and FRAP results were in a similar range (3592 to 5454 µmol TE/L and 3089 to 5261 µmol TE/L, respectively). On both assays, the AOC of the PC sample was significantly lower than other samples, while the ST, BT and BW samples showed the highest AOC. The FRAP results of the wine samples were comparable to that of other polyphenol-rich beverages (pomegranate juice, red wine, grape juice, blueberry juice, black cherry juice, acai juice, cranberry juice, with concentrations of 3400 to 4900 µmol TE/L by FRAP), and much higher than other frequently consumed beverages (orange juice, iced green tea, iced black tea, iced white tea, apple juice, with concentrations of 500 to 1700 µmol TE/L) [34]. This indicates that the cranberry wine could be a competitive product based on market interest in bioactive compounds as added functional benefit in food and beverages.

The TPC (Figure 1A) of the Stevens and Bergman wines (with the range of 173 to 239 mg/L) showed similar results, were both higher than the Pilgrim samples (with a range of 139 to 212 mg/L), and inconsistent with the TPC of juice samples. Comparing the vinification approaches, the White samples showed less advantage in terms of TPC compared to the Thermo and Red samples. Similarly, this is also true for the ANC (Figure 1B), where the range is from 23.6 to 50.9 mg/L. These findings are in agreement with the red wine study, resulting in similar TPC and ANC of wine samples fermented through traditional red vinification and thermovinification [18].

PAC values (Figure 1C) were also obtained for the three cultivars, which is in the same sequence as in the juices: Bergman (224 to 293 mg/L) > Stevens (192 to 277 mg/L) > Pilgrim (129 to 210 mg/L). The values were close to those obtained for the total phenolics, suggesting the presence of proanthocyanins as the major phenolic species present. The PAC values from the three vinifications (Red > Thermo > White) showed the enhanced PAC extraction by Red and Thermo methods, indicating pomace (including skin and seeds) may be a good source of PAC. Skin and seeds were demonstrated to contain a high amount of PAC in previous studies [7]. It has been reported that there are high amounts of cell wall-bound PACs in cranberry that are resistant to common extraction, meaning that the PAC contents can be underrated [35]. The increased amount of PACs in the wines may originate from these cell-wall bound PACs, which were better extracted during heat-treatment (Thermo method) and from pomace during fermentation.

Among the three cultivars, Pilgrim wine contains the lowest amount of polyphenols and AOC, indicating Stevens and Bergman cultivars as the more suitable cranberry varieties for winemaking in the interest of achieving higher polyphenol content in the final product. Santos, et al. [36] studied the bioactivity and sensory quality of Brazilian blueberry wine, and found a positive effect of phenolic compounds on wine flavour, as the samples with higher phenolic compounds were correlated with higher preference in sensory. If there is an optimized phenolic content range for good sensory evaluation is still unclear, which needs to be investigated in our further study.

### 3.2. Identification and Quantification of Polyphenols in Cranberry Wine

Qualification and quantification of the anthocyanins were carried out using HPLC according to Section 2.5. Qualification of phenolic acids, flavonols, and proanthocyanins was undertaken on LC-MS/MS by comparing the retention times, along with MS and MS/MS data, with available standards. A list of the identified compounds is presented in Table S1. The concentration of the polyphenols in nine cranberry wines is presented in Table 2. Four compounds (*m*-salicylic acid, vanillic acid, trans-sinapic acid and kaempferol) were present in trace amounts in the wine samples, and their contents were not quantified (Table 2). Overall, results show that ANC, PAC, flavonol and phenolic acids were the major classes of phenolics present in the cranberry wine samples. Among the four classes identified, anthocyanins accounted for the largest proportion (28% to 47%), while the other classes made up lower proportions: proanthocyanins (11% to 30%), phenolic acids (17% to 23%), and flavonols (20 to 34%). 

The results of total ANC from the HPLC analysis of the wines (Table 2A) were in agreement with the spectrophotometric measurement method, where Stevens had the highest levels among the three cultivars. Comparing the vinification method, thermovinification had a better advantage than the White and Red approaches, revealing that heat treatment at 70 °C increased the ANC extraction from pomace. The ANC content is the compositive result of extraction, thermal and enzyme degradation. From the literature, high temperatures were applied regularly to advance extraction efficiency as it improves solvent penetration, compound diffusion, and solubility [37,38,39]. Furthermore, heat treatment could also enhance anthocyanin extraction by inhibiting enzymatic oxidation. Nonetheless, anthocyanins have been reported as being heat-sensitive, and a high temperature could cause their degradation [39]. Thus, there is an ideal temperature expected for different plant matrices, depending on their anthocyanin composition. The previous study on blueberry anthocyanins indicated that a temperature higher than 80 °C could affect stability [40]. Our current study results revealed that 70 °C was a suitable temperature for producing cranberry wine to give high ANC, as it enhanced extraction without adversely affecting the ANC composition (compared to cranberry juice, unpublished data). Among the six anthocyanins determined, cynidin-3-arabinoside present at the highest amount (44.9% to 72.2%), while cynidin-3-glucoside occupied the smallest proportion (1.2% to 3.2%), similar to the cranberry anthocyanin composition in the literature [29].

Results of total PAC obtained from LC-MS/MS (10.3 to 37.0 mg/L) (Table 2B) were much lower than those from the DMAC method (129 to 293 mg PAC A2 equivalent/L) (Figure 1). This dissimilarity is caused by that the profiling of proanthocyanins by LC-MS/MS only included monomers and dimers owing to the available commercial standards, which consisted only a small portion of total PACs. As reported in other studies, PACs present mainly as polymers in cranberries, with an average polymerization of 15 flavonoid units [4]. Considering cultivar effects, total PAC (25.1 to 37.0 mg/L) and all factions in PAC showed the highest amount in the Bergman samples, while the Pilgrim samples contained the lowest PAC (10.3 to 13.8 mg/L), in agreement with the DMAC method (Figure 1). The highest PAC appeared in the BW sample (37.0 mg/L), 3.6 times greater than that in the PT sample (10.3 mg/L). The PAC results of wines share a similar pattern with juices among cultivars (Table 1), indicating cultivar difference as a potential reason for this diversity. In contrast, different treatments did not give significant difference in PAC (*p* > 0.05). Of PAC monomers and dimers, epicatechin accounted for the highest amount (39.6% to 46.5%), and the proportion of epicatechin is similar among all samples.

Although phenolic acids were higher in the Bergman samples (20.4 to 23.1 mg/L) than the Stevens and Pilgrim samples (16.0 to 21.5 mg/L), the difference was not significant (*p* > 0.05) (Table 2C). Similarly, different vinification approaches did not contribute to any significant difference in the total content of phenolic acids (*p* > 0.05). The main phenolic acids were: *p*-coumaric acid (40.7% to 56.8%) > protocatechuic acid (14.5% to 30.1%) > caffeic acid (9.7% to 12.7%) > chlorogenic acid (3.5% to 16.7%). β-resorcylic acid was only detected in the PR sample.

The three cultivars did not show a significant difference in the flavonol content (*p* > 0.05) (Table 2D). However, the Thermo method (32.1 to 34.2 mg/L) led to significantly higher amounts of flavonols than the White (20.8 to 25.9 mg/L) and Red (18.0 to 18.6 mg/L) methods, indicating heat treatment enhanced the extraction of flavonols. This finding is supported by the previous study reporting the improved extraction of quercetin from onion skin when extraction temperature increased [41]. Among all of the flavonols identified, quercetin (40.4% to 71.4%) and quercetin-3-rhamnoside (17.84–35.00%) were the major compounds present in the wine samples. However, quercetin-3-galactoside was known as the most prevalent form of flavonol in cranberry fruit, which accounted for about 75% of total flavonols [42]. Current results thus implied that this compound was degraded to quercetin during fermentation.

### 3.3. Distribution of Phenolic Compounds in Different Samples

**Figure 2:**
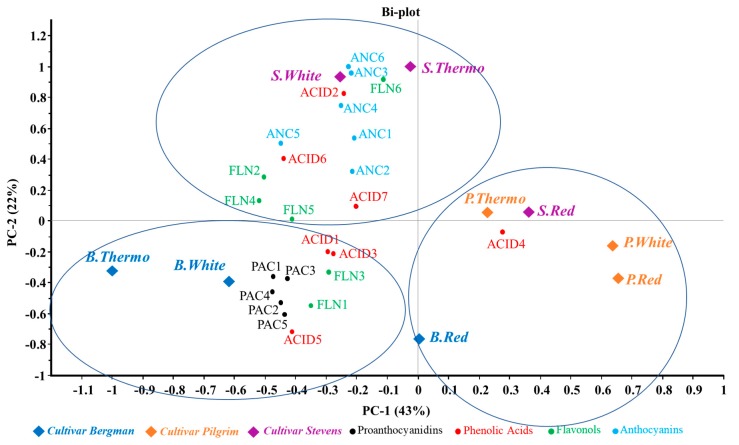
Principle component analysis (PCA) of phenolic compounds. The compound names were represented by code (PAC: proanthocyanins, ACID: phenolic acids, FLN: flavonols, ANC: anthocyanins; PC-1: 1st principal component; PC-2: 2nd principal component; B.Thermo: Thermovinification; B.White: White vinification; B.Red: Red vinification; S.White: Stevens White; S.Thermo: Stevens Thermo; S.Red: Stevens Red; P.Thermo: Pilgrim Thermo; P.White: Pilgrim White; P.Red: Pilgrim Red; B.Thermo: Bergman Thermo; B.White: Bergman White; B.Red: Bergman Red

The PCA on the phenolic compounds content of the wine samples is shown in Figure 2. Different chemical classes of compounds are indicated by different colours in Figure 2. Results show that the two main principal compounds (PCs) accounted for 67% of the total variability in the nine wine samples, with PC1 accounting for 43% and PC2 for 22%. The samples appeared to be divided into three clusters in the PCA map. Among these samples, the SW and ST samples were positively correlated with anthocyanins, and some of the flavonols and phenolic acids. The BW and BT samples were associated with higher concentrations of proanthocyanins, and some of the phenolic acids and flavonols. However, all Pilgrim samples and all Red samples were not correlated with most of the detected polyphenols. A notable difference in phenolic composition of the wines from the three cultivars revealed Bergman and Stevens as the more favourable cultivars for winemaking than Pilgrim.

Among the three vinification methods, White and Thermo are more correlated with the identified monomeric phenolic compounds. However, taking the TPC results into consideration, the White samples showed the lowest TPC among all vinification methods, indicating that the White samples have more monomeric phenolics than can be measured by HPLC, but consistently have less oligomeric and polymeric forms. The oligomer and polymers are potentially from the contact with pomace (through Red and Thermo). Overall, the results revealed improved extraction of both phenolic monomers and polymers by the Thermo treatment.

### 3.4. Quantitative Analysis of Volatile Compounds

A total of 31 volatile compounds were quantified in the nine wine samples (Table 3). The profile includes 14 esters, six alcohols, five acids, and six others. Esters were found as the most diversified group, while both alcohol and acids were present in abundance. The compounds that primarily contributed to the profiles were: ethyl acetate (4106 to 6904 µg/L), ethyl benzoate (1631 to 4928 µg/L), isobutanol (7515 to 16,475 µg/L), isoamyl alcohol (23,904 to 30,073 µg/L), benzyl alcohol (753 to 2604 µg/L), phenylethyl alcohol (2752 to 4108 µg/L), and benzoic acid (47,267 to 72,760 µg/L).

The effects of the factors (cultivar and vinification) on volatiles were analysed by the two-way ANOVA (Table 3), showing vinification had an impact on 23 out of 31 compounds, while cultivar had a significant effect on 18 out of 31 compounds, indicating vinification methods have more influence on volatiles than cultivars. The results also show some of the identified compounds (isobutyl acetate, ethyl methylbutyrate and pentanal) with no significant difference in concentrations (*p* > 0.05), regardless of cultivars or vinification methods.

The contribution of one specific volatile compound to the perception of the aroma depends not only on the concentration of the volatile compound itself but also on its odour threshold value. Regarding this, we examined the threshold of the compounds and listed the aroma-active volatiles in Table 4 (OAVs > 1). From the analysis, 13 volatiles potentially made the highest contribution to the aroma profile of the samples with OAVs > 10, including phenylethyl alcohol (OAV: 183453–273855), β-damascenone (OAV: 1069–7085), benzyl alcohol (OAV: 627–2170), ethyl methylbutyrate (OAV: 785–1324), ethyl acetate (OAV: 821–1381), ethyl butyrate (OAV: 304–531), ethyl hexanoate (OAV: 42-141), isoamylalcohol (OAV: 96–116), methylbutyric acid (OAV: 29–54), ethyl benzoate (OAV: 16–49), isobutanol (OAV: 21–46), geraniol (OAV: 15–24) and α-terpineol (OAV: 8–17). These volatiles were responsible for the fruity and floral aroma (Table 4).

Except for β-damascenone and geraniol, 11 out of the above 13 aroma-active volatiles have been reported to be present in cranberry fruits [43,44], indicating they are cranberry-derived compounds contributing to the characteristic cranberry aroma. Despite these compounds being present in both the cranberry fruit and wines, their role in aroma perception is not the same. Taking the most prevalent aroma contributors as an example, namely phenylethyl alcohol, benzyl alcohol, ethyl methylbutyrate, and ethyl acetate, although they were identified as the main fruity and floral aroma contributors in cranberry wines with the OAVs from 627 to 273855, they were not crucial volatile contributors to the aroma of cranberry fruit with OAVs as low as < 1 in the literature [43]. The discrepancy of OAVs for the above compounds indicates that the compounds may originate from both fruit and wine fermentation. However, the contribution from the fermentation should be the major one as the OAVs were much higher in wine samples. It is known that the main aromas (esters and alcohols) in wine are generally produced through the yeast pathways with esters produced from lipid metabolism, and fusel alcohols from the synthesis of amino acids and proteins [45].

Though β-damascenone was rarely reported from cranberry, it has been well studied in red wine as a major contributor for rose aroma, despite its low concentration (1–1.5 µg/L in red wine) [17]. In red wine, β-damascenone is a grape-derived compound, as found in grape skins. It is produced from β-carotene during ripening of grapes. However, its presence and pathway in cranberry wines have never been reported in previous pieces of literature. Similarly, geraniol is also a grape-derived volatile compound generated in the skin. The amount of geraniol in red wine was reported to decrease by heat treatment of 75 °C [17], which was not observed in Thermovinification at 70 °C in our study. Geraniol was found to be present in the cranberry must at a higher level than in the wines (unpublished data), indicating geraniol as a cranberry-derived compound.

### 3.5. Distribution of Volatile Compounds in Different Samples

**Figure 3:**
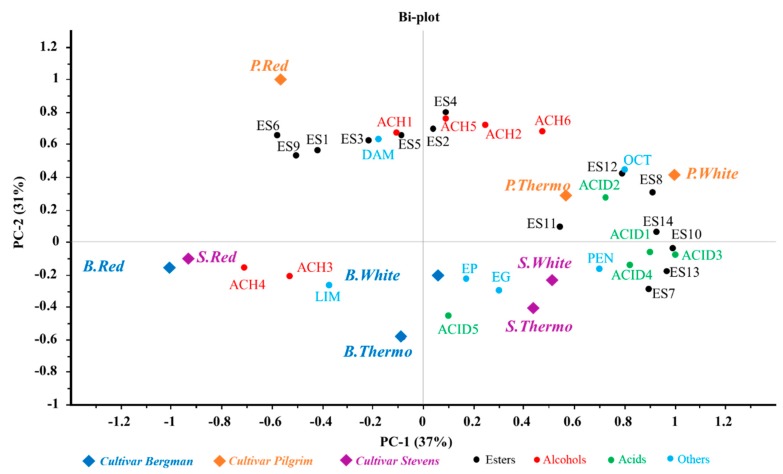
Principle component analysis (PCA) volatile compounds in cranberry wines. ACH: alcohol; LIM: D-Limonene; ES: ester; DAM: β-damascenone; EP: 4-ethylphenol; EG: 4-ethylguaiacol; OCT: 3-Octanone; PEN: Pentanal.

PCA was constructed to give further insight into the relationship between samples and volatiles, based on the average concentrations of the volatiles in the samples. Results (Figure 3) showed that PC1 and PC2 are capable of explaining 37% and 31% of the variation in data, respectively. The result from PCA divided the samples into four clusters. The BW, BT, BR and SR samples were located in the lower left quadrant, showing no correlation with most of the volatiles, except for two alcohols and one terpene. The SW and ST samples were in the right lower quadrant, showing strong association with some esters, phenols and acids. The PR sample was located in the upper left quadrant, showing a high concentration of five esters and one alcohol. The PW and PT samples were in the upper right cluster, with strong correlations with three alcohols, seven esters, and two acids. According to the results, samples with different treatments and cultivars were well distinguished, revealing the effects of both factors on the volatile profiles.

Looking at the different cultivars, the Stevens and Bergman samples occupied the lower side of the map, while the Pilgrim samples appeared in the upper quadrant, showing more correlation with most of the esters and some alcohols. Meanwhile, nearly all aroma-active volatiles showed a higher content in the Pilgrim samples than the Bergman and Stevens samples (Table 4). The Pilgrim cultivar was found to be more favourable for winemaking than the Stevens and Bergman ones, as it produces a higher level of volatiles, which is responsible for desirable fruity and floral aroma. The diversity of volatile profiles of samples is believed to be derived from both the cranberry and the fermentation process. The cranberry-derived volatiles may be correlated to their original contents in the cranberry musts of different cultivars, which will be confirmed in further study. The fermentation-derived volatiles may be related to chemical compositions of musts (such as phenolics), which may influence the volatile production during fermentation. According to a study on red wine [46], the production of 4-ethylphenol, a volatile metabolic product of fermentation, decreased by adding polyphenolic compounds, especially procyanidic tannins. In agreement with the above study, the concentration of the 4-ethylphenol compound was also low in the Bergman wines in the current study, which had the highest total PACs and individual PAC monomers and dimers. Similarly, another study demonstrated the degradation and inhibitory effects of phenol monomers on the production of aromatic compounds [47]. In accordance with the above studies, our findings revealed the potential inhibitory effects of phenolic compounds on volatile production, especially PACs.

Considering the vinification approaches, the Red samples were located in the left side of the map, while the Thermo and White samples were in the right quadrant, showing increasing correlation with more volatiles produced, indicating that the White and Thermo methods are more beneficial than the Red method. The vinifications methods may have impacts on the degradation of cranberry-derived volatiles (mostly related to the Thermo method), and generation of fermentation-derived volatiles. The limitation of volatile production during Red vinification may be correlated with the high amount of PACs in musts, which was possibly due to the presence of the pomace during fermentation.

## 4. Conclusions

This research investigated the influence of cultivars and vinification methods on cranberry wine fermentation. Results showed that the thermovinification is more advantageous on cranberry winemaking than the traditional Red and White vinifications by enhancing both phenolic (monomeric and polymeric) and volatile compositions. The phenolic and volatile compositions of the wines are highly correlated with that of fruit cultivars. Higher phenolic contents in musts resulted in higher phenolic retention in wines. On the other hand, cultivars with a higher amount of polyphenols (especially PACs) produce relatively fewer volatiles after fermentation. Concerning both phenolics and volatiles as significant factors on wine quality, a fruit cultivar with high phenolic content but relatively low PAC content is preferred (such as Stevens in this study). This is the first study on phenolic and volatile profiles of cranberry wines, it could be a reference for vinification and cultivar selection for cranberry wine specifically, and also for phenol-rich fruit wine production in general.

## Figures and Tables

**Table 1:** Total phenolic (TPC), anthocyanin (ANC), proanthocyanin (PAC) contents and antioxidant capacities (AOC) of the juice samples determined by spectrophotometric assays.

Sample	TPC ^A^	Total ANC ^B^	Total PAC ^C^	Total AOC ^D^		pH	Brix (°)
				FRAP	ABTS		
***Stevens***	882.0 ± 2.5 ^b^	283.3 ± 21.3 ^a^	445.8 ± 22.2 ^b^	9770.0 ± 563.0 ^a^	9506.9 ± 21.3 ^a^	2.85 ± 0.04 ^a^	5.33 ± 0.46 ^a^
***Pilgrim***	660.1 ± 12.5 ^c^	168.1 ± 9.8 ^c^	397.7 ± 21.1 ^c^	7470.0 ± 264.6 ^b^	8429.5 ± 307.4 ^b^	2.98 ± 0.15 ^a^	5.20 ± 0.20 ^a^
***Bergman***	938.7 ± 10.3 ^a^	210.7 ± 5.9 ^b^	583.9 ± 12.8 ^a^	9908.9 ± 333.9 ^a^	9557.9 ± 24.1 ^a^	2.99 ± 0.18 ^a^	5.43 ± 0.32 ^a^

^A^ TPC expressed as mg gallic acid equivalent (GAE) per L; ^B^ ANC expressed as mg Cyn-Glu equivalent per L; ^C^ PAC expressed as mg PAC A2 equivalent per L; ^D^ Total AOC expressed as µmol Trolox equivalent (TE) per L. Values identified by the same letters are not significantly different at the 0.05 level (One way ANOVA, Tukey^ab^). FRAP: ferric reducing ability of plasma method; ABTS: free radical-scavenging activity method.

**Table 2:** The concentration of phenolic compounds detected in cranberry wines by HPLC (high-performance liquid chromatography) (anthocyanins) and LC-MS/MS (liquid chromatography technique coupled with tandem mass spectrometry) (proanthocyanins, phenolic acids and flavonols).

	RT (min)	Compounds	Concentration (µg/mL)										
			Stevens				Pilgrim				Bergman		
			Clear	Thermo	Whole		Clear	Thermo	Whole		Clear	Thermo	Whole
A		***Anthocyanins***											
	7.39	Cyanidin 3-galactoside	2.50 ± 0.21 ^b^	2.20 ± 0.16 ^b^	–		1.17 ± 0.02 ^c^	3.86 ± 0.05 ^a^	0.20 ± 0.01 ^d^		1.34 ± 0.03 ^c^	2.45 ± 0.38 ^b^	0.26 ± 0.12 ^d^
	8.07	Cyanidin 3-glucoside	0.85 ± 0.07 ^ab^	0.77 ± 0.00 ^abc^	0.95 ± 0.28 ^a^		0.31 ± 0.01 ^c^	0.41 ± 0.06 ^bc^	0.71 ± 0.02 ^abc^		0.61 ± 0.04 ^abc^	0.86 ± 0.16 ^ab^	0.65 ± 0.15 ^abc^
	8.54	Cyanidin 3-arabinoside	28.77 ± 1.49 ^a^	28.70 ± 0.58 ^a^	22.26 ± 3.22 ^ab^		13.11 ± 0.47 ^b^	15.88 ± 0.40 ^bcd^	17.23 ± 0.40 ^bcd^		18.92 ± 0.09 ^bcd^	21.98 ± 2.77 ^abc^	14.53 ± 3.34 ^cd^
	9.18	Peonidin 3-galactoside	5.83 ± 0.16 ^a^	4.81 ± 0.18 ^bc^	0.24 ± 0.08 ^e^		2.54 ± 0.14 ^c^	5.52 ± 0.04 ^ab^	0.38 ± 0.10 ^e^		3.06 ± 0.09 ^d^	4.43 ± 0.50 ^c^	0.20 ± 0.14 ^e^
	9.78	Peonidin 3-glucoside	4.31 ± 0.09 ^ab^	3.51 ± 0.10 ^abc^	2.78 ± 0.50 ^bcd^		1.76 ± 0.07 ^b^	1.74 ± 0.05 ^d^	2.01 ± 0.39 ^d^		3.82 ± 0.05 ^ab^	4.09 ± 0.49 ^ab^	2.17 ± 0.72 ^cd^
	10.22	Peonidin 3-arabinoside	15.89 ± 0.89 ^a^	15.43 ± 0.22 ^a^	10.79 ± 1.47 ^bc^		7.23 ± 0.31 ^bc^	7.99 ± 0.24 ^bcd^	8.17 ± 0.56 ^bcd^		10.26 ± 0.01 ^bcd^	11.27 ± 1.14 ^b^	6.68 ± 1.70 ^d^
		**Total**	**58.15 ± 2.03 ^a^**	**55.42 ± 0.56 ^a^**	**37.03 ± 5.53 ^bc^**		**26.12 ± 1.01 ^c^**	**35.40 ± 0.54 ^bc^**	**28.69 ± 1.24 ^c^**		**38.02 ± 0.11 ^bc^**	**45.09 ± 5.44 ^ab^**	**24.50 ± 6.18 ^c^**
B		***Proanthocyanidins***											
	3.51	PAC B1	0.75 ± 0.14 ^a^	0.29 ± 0.01 ^a^	0.38 ± 0.07 ^a^		0.38 ± 0.04 ^a^	0.20 ± 0.05 ^a^	0.33 ± 0.17 ^a^		1.10 ± 0.35 ^a^	1.12 ± 0.40 ^a^	0.71 ± 0.41 ^a^
	4.68	Catechin	2.63 ± 0.03 ^abc^	1.73 ± 0.04 ^c^	1.70 ± 0.20 ^c^		2.12 ± 0.30 ^bc^	1.29 ± 0.20 ^c^	2.12 ± 0.66 ^bc^		4.19 ± 0.88 ^ab^	4.74 ± 0.57 ^a^	3.33 ± 0.92 ^abc^
	6.30	PAC B2	3.74 ± 0.29 ^abc^	1.44 ± 0.10 ^bc^	1.73 ± 0.51 ^bc^		1.51 ± 0.02 ^bc^	0.88 ± 0.04 ^c^	1.87 ± 0.52 ^bc^		4.74 ± 1.63 ^ab^	5.94 ± 1.86 ^a^	3.56 ± 1.39 ^abc^
	7.61	Epicatechin	8.41 ± 0.60 ^bc^	6.34 ± 0.30 ^c^	5.77 ± 0.77 ^c^		5.79 ± 0.56 ^c^	4.47 ± 0.31 ^c^	5.48 ± 1.65 ^c^		16.27 ± 2.94 ^a^	15.48 ± 2.37 ^ab^	11.24 ± 3.83 ^abc^
	11.52	PAC A2	3.70 ± 0.11 ^bc^	3.83 ± 0.09 ^bc^	3.80 ± 0.30 ^bc^		3.08 ± 0.04 ^c^	3.50 ± 0.38 ^bc^	4.00 ± 0.02 ^bc^		5.87 ± 1.41 ^ab^	7.88 ± 1.10 ^a^	6.25 ± 0.95 ^ab^
		**Total**	**19.24 ± 0.96 ^abc^**	**13.62 ± 0.33 ^c^**	**13.39 ± 1.85 ^c^**		**12.89 ± 0.88 ^c^**	**10.33 ± 0.22 ^c^**	**13.80 ± 3.03 ^bc^**		**37.01 ± 12.42 ^a^**	**35.16 ± 6.29 ^ab^**	**25.08 ± 7.50 ^abc^**
C		***Phenolic Acids***											
	1.28	Gallic acid	0.41 ± 0.11 ^bcde^	0.37 ± 0.04 ^bcd^	0.35 ± 0.01 ^bcd^		0.30 ± 0.03 ^bcde^	0.20 ± 0.01 ^de^	0.12 ± 0.01 ^e^		2.06 ± 0.07 ^a^	0.48 ± 0.06 ^b^	0.25 ± 0.01 ^cde^
	2.16	Protocatechuic acid	6.46 ± 0.89 ^a^	5.47 ± 0.05 ^ab^	4.61 ± 0.04 ^bc^		4.44 ± 0.00 ^bc^	3.64 ± 0.28 ^cd^	3.21 ± 0.40 ^cd^		5.62 ± 0.05 ^ab^	4.52 ± 0.09 ^bc^	2.96 ± 0.39 ^d^
	4.59	Gentisic acid	0.89 ± 0.01 ^ab^	0.89 ± 0.01 ^ab^	0.94 ± 0.02 ^ab^		0.75 ± 0.05 ^b^	0.83 ± 0.01 ^ab^	0.86 ± 0.07 ^ab^		0.88 ± 0.10 ^ab^	0.95 ± 0.02 ^ab^	1.00 ± 0.05 ^ab^
	5.09	*β*-Resorcylic acid	−	−	−		−	−	0.43 ± 0.19 ^a^		−	−	−
	5.21	Chlorogenic acid	0.87 ± 0.08 ^bc^	0.74 ± 0.12 ^c^	1.04 ± 0.10 ^bc^		0.97 ± 0.42 ^bc^	1.90 ± 0.31 ^ab^	1.14 ± 0.03 ^bc^		2.80 ± 1.07 ^ab^	3.95 ± 0.85 ^a^	2.64 ± 0.48 ^abc^
	5.71	Caffeic acid	2.72 ± 0.28 ^a^	2.35 ± 0.03 ^a^	2.05 ± 0.10 ^a^		1.87 ± 0.24 ^a^	1.87 ± 0.40 ^a^	1.80 ± 0.01 ^a^		2.32 ± 0.00 ^a^	2.55 ± 0.19 ^a^	2.34 ± 0.44 ^a^
	8.45	*p*-Coumaric acid	10.11 ± 0.16 ^ab^	10.99 ± 0.35 ^ab^	8.83 ± 0.43 ^ab^		10.93 ± 0.59 ^ab^	10.21 ± 0.52 ^ab^	8.42 ± 0.86 ^b^		9.38 ± 0.36 ^ab^	11.24 ± 0.39 ^a^	11.19 ± 1.56 ^ab^
		**Total**	**21.47 ± 1.35 ^abc^**	**20.86 ± 0.48 ^abc^**	**17.94 ± 0.78 ^bc^**		**19.26 ± 1.32 ^abc^**	**18.64 ± 1.51 ^abc^**	**15.98 ± 0.69 ^c^**		**23.06 ± 1.51 ^ab^**	**23.69 ± 0.67 ^a^**	**20.37 ± 2.91 ^abc^**
D		***Flavonols***											
	9.86	Myricetin-3-galactoside	1.08 ± 0.04 ^d^	1.44 ± 0.01 ^d^	1.62 ± 0.07 ^d^		1.53 ± 0.23 ^d^	4.52 ± 0.06 ^b^	2.46 ± 0.07 ^cd^		3.82 ± 1.04 ^bc^	7.02 ± 0.75 ^a^	2.76 ± 0.26 ^bcd^
	10.09	Myricetin-3-glucoside	0.47 ± 0.05 ^ab^	0.48 ± 0.02 ^ab^	0.40 ± 0.04 ^ab^		0.31 ± 0.03 ^b^	0.45 ± 0.06 ^ab^	0.31 ± 0.01 ^b^		0.49 ± 0.15 ^ab^	0.61 ± 0.05 ^ab^	0.38 ± 0.02 ^ab^
	11.39	Quercetin-3-galactoside	0.17 ± 0.03 ^c^	0.20 ± 0.00 ^c^	0.07 ± 0.01 ^c^		0.69 ± 0.08 ^bc^	3.27 ± 0.21 ^a^	0.46 ± 0.14 ^c^		1.69 ± 0.60 ^b^	3.78 ± 0.41 ^a^	0.34 ± 0.16 ^c^
	12.90	Quercetin-3-rhamnoside	6.56 ± 0.28 ^ab^	6.64 ± 0.03 ^ab^	5.88 ± 0.13 ^ab^		5.06 ± 0.47 ^b^	5.79 ± 0.93 ^ab^	5.60 ± 0.19 ^ab^		6.68 ± 0.80 ^ab^	7.63 ± 0.62 ^a^	6.31 ± 0.74 ^ab^
	14.76	Phloridzin	0.93 ± 0.00 ^a^	1.00 ± 0.03 ^a^	0.84 ± 0.03 ^a^		0.78 ± 0.06 ^a^	1.13 ± 0.18 ^a^	0.82 ± 0.02 ^a^		1.02 ± 0.40 ^a^	1.17 ± 0.27 ^a^	0.96 ± 0.15 ^a^
	18.68	Quercetin	15.38 ± 1.88 ^bcd^	24.37 ± 0.10 ^a^	9.58 ± 0.05 ^def^		12.39 ± 0.03 ^cde^	17.31 ± 0.53 ^bcd^	8.90 ± 1.55 ^ef^		12.18 ± 1.45 ^cde^	13.95 ± 1.37 ^bcd^	7.29 ± 1.33 ^f^
		**Total**	**24.59 ± 2.28 ^bc^**	**34.13 ± 0.08 ^a^**	**18.38 ± 0.11 ^c^**		**20.75 ± 0.85 ^c^**	**32.49 ± 0.92 ^ab^**	**18.55 ± 1.94 ^c^**		**25.87 ± 4.42 ^abc^**	**34.16 ± 3.47 ^a^**	**18.02 ± 2.66 ^c^**

(–) not detected. PAC B1, PAC B2, PAC A2 refers to Procyanidin B1, B2, and A2, respectively. (RT) retention time. Data are presented as mean ± SD. Values identified by the same letters are not significantly different at the 0.05 level (Tukey-Kramer HSD test).

**Table 3:** Concentration of volatile compounds detected in cranberry wines by GC-MS (gas chromatography combined with mass spectrometry).

No.	Compound	CAS	Codes	Concentration (µg/L)																				*p*-value	
				Stevens							Pilgrim							Bergman							
				White	*SD%*	Thermo	*SD%*	Red	*SD%*		White	*SD%*	Thermo	*SD%*	Red	*SD%*		White	*SD%*	Thermo	*SD%*	Red	*SD%*	Cultivar	Vinification
***Esters***																									
1	Ethyl Acetate	141-78-6	ES1	5,716 ^ab^	11	4811 ^bc^	3	5,648 ^ab^	9		5714 ^ab^	2	4,929 ^bc^	9	6904 ^a^	6		5582 ^abc^	4	4106 ^c^	17	6345 ^a^	5	0.1	<0.0001 *
2	Methyl butanoate	623-42-7	ES2	0.72 ^de^	13	0.52 ^e^	12	0.49 ^e^	9		1.62 ^b^	6	1.17 ^c^	3	2.15 ^a^	7		1.16 ^c^	11	1.00 ^cd^	23	1.08 ^cd^	16	<0.0001 *	0.0223 *
3	Isobutyl acetate	110-19-0	ES3	10.02 ^a^	12	10.45 ^a^	2	11.00 ^a^	4		10.75 ^a^	2	11.23 ^a^	4	11.33 ^a^	6		10.55 ^a^	16	9.35 ^a^	2	10.81 ^a^	2	0.1	0.3
4	Ethyl butyrate	105-54-4	ES4	35.76 ^bc^	9	33.55 ^bc^	4	38.97 ^abc^	7		44.96 ^abc^	13	46.93 ^ab^	11	53.08 ^a^	14		39.04 ^abc^	24	30.40 ^c^	5	34.47 ^bc^	3	<0.0001 *	0.2
5	Ethyl methylbutyrate	7452-79-1	ES5	11.09 ^a^	6	9.11 ^a^	13	11.65 ^a^	7		10.71 ^a^	20	11.53 ^a^	23	13.24 ^a^	26		11.27 ^a^	31	7.85 ^a^	28	9.43 ^a^	13	0.2	0.3
6	Isoamyl acetate	123-92-2	ES6	27.89 ^ab^	15	25.71 ^c^	3	38.49 ^b^	5		32.70 ^bc^	7	31.05 ^bc^	7	58.39 ^a^	17		29.65 ^bc^	15	26.45 ^bc^	5	40.14 ^b^	2	0.0108 *	0.0011 *
7	Methyl hexanoate	106-70-7	ES7	7.87 ^ab^	3	8.39 ^a^	3	4.37 ^d^	3		8.44 ^a^	5	7.54 ^ab^	10	4.86 ^d^	8		6.58 ^bc^	11	8.14 ^a^	1	5.23 ^cd^	7	0.9	<0.0001 *
8	Ethyl hexanoate	123-66-0	ES8	35.86 ^ab^	18	29.70 ^bc^	12	16.40 ^d^	2		42.18 ^a^	11	37.85 ^ab^	2	28.44 ^bc^	13		22.95 ^cd^	19	22.48 ^cd^	6	12.66 ^d^	3	<0.0001 *	<0.0001 *
9	Methyl benzoate	93-58-3	ES9	83.25 ^bcd^	11	57.52 ^cd^	14	101.03 ^abc^	15		106.65 ^ab^	15	48.11 ^d^	4	145.55 ^a^	20		74.95 ^bcd^	5	69.83 ^bcd^	20	110.20 ^ab^	23	0.2	0.0003 *
10	Ethyl octanoate	106-32-1	ES10	43.79 ^ab^	5	34.94 ^bc^	14	6.25 ^e^	7		49.37 ^a^	18	40.39 ^abc^	10	14.88 ^de^	18		26.67 ^cd^	18	31.18 ^bc^	5	4.98 ^e^	4	0.1	<0.0001 *
11	Ethyl benzoate	93-89-0	ES11	3,752 ^ab^	11	2,701 ^bc^	16	1631 ^c^	20		4,928 ^a^	17	1,730 ^c^	7	3122 ^bc^	18		4,128 ^ab^	23	3068 ^bc^	17	2412 ^bc^	26	0.4	<0.0001*
12	β-phenylethyl acetate	103-45-7	ES12	267.79 ^a^	2	262.27 ^ab^	0	252.14 ^b^	2		271.28 ^a^	1	271.15 ^a^	2	267.32 ^a^	3		263.57 ^ab^	2	260.15 ^ab^	0	257.77 ^ab^	1	0.0049 *	0.0275*
13	Ethyl decanoate	110-38-3	ES13	5.18 ^a^	8	4.43 ^a^	9	1.82 ^b^	1		5.03 ^a^	5	4.80 ^a^	15	2.16 ^b^	9		4.15 ^a^	1	4.09 ^a^	18	1.80 ^b^	2	0.5	<0.0001*
14	Ethyl cinnamate (*trans*)	103-36-6	ES14	93.11 ^b^	10	99.84 ^b^	20	22.73 ^f^	7		170.71 ^a^	4	83.96 ^bc^	13	36.94 ^def^	7		62.05 ^cd^	2	59.35 ^cde^	13	27.83 ^ef^	5	0.1	0.0011 *
***Alcohols***																									
15	Isobutanol	78-83-1	ACH1	11,764 ^bc^	22	11,797 ^bc^	7	13,601 ^ab^	3		15,844 ^a^	3	15,020 ^ab^	11	16,475 ^a^	5		11,010 ^bc^	17	7,515 ^c^	8	15,331 ^ab^	9	<0.0001 *	0.0044 *
16	Isoamylalcohol	123-51-3	ACH2	26,280 ^a^	14	25,705 ^a^	6	26,720 ^a^	3		28,911 ^a^	3	26,997 ^a^	6	30,073 ^a^	2		25,320 ^a^	7	25,281 ^a^	18	23,904 ^a^	11	0.0087 *	0.5
17	α-terpineol	98-55-5	ACH3	288.42 ^bc^	10	228.89 ^c^	6	293.06 ^b^	7		286.13 ^bc^	3	272.78 ^bc^	4	319.28 ^b^	5		423.71 ^a^	11	415.03 ^a^	3	473.69 ^a^	0	<0.0001 *	0.0006 *
18	Benzyl alcohol	100-51-6	ACH4	1,842 ^ab^	4	1768 ^b^	9	2604 ^a^	2		918 ^cd^	4	752.67 ^d^	4	1,843 ^a^	13		1181 ^bcd^	31	1527 ^bcd^	10	1785 ^abc^	7	0.0002 *	0.0007 *
19	Geraniol (*trans*)	106-24-1	ACH5	68.50 ^a^	1	58.97 ^a^	16	64.67 ^a^	10		84.70 ^a^	14	85.71 ^a^	15	95.74^a^	23		75.40 ^a^	33	65.46 ^a^	26	73.03 ^a^	14	0.0084 *	0.5
20	Phenylethyl alcohol	60-12-8	ACH6	3,116 ^bc^	13	2904 ^c^	8	2859 ^c^	5		4108 ^a^	2	3,308 ^bc^	8	3778^ab^	6		2990 ^bc^	5	2775 ^c^	14	2752 ^c^	7	<0.0001 *	0.0225 *
***Acids***																									
21	Methylbutyric acid	116-53-0	ACID1	523.23 ^ab^	10	429.26 ^abcde^	21	292.55 ^e^	6		541.72 ^a^	5	456.33 ^abcd^	13	317.79 ^de^	17		496.39 ^abc^	2	366.73 ^bcde^	15	330.94 ^cde^	1	0.9	0.0001 *
22	Hexanoic acid	142-62-1	ACID2	306.54 ^cde^	6	475.47 ^ab^	16	272.14 ^de^	6		451.20 ^abc^	8	579.65 ^a^	12	345.68 ^bcd^	12		300.40 ^cde^	33	255.80 ^de^	2	178.86 ^e^	11	<0.0001 *	0.0001 *
23	Octanoic acid	124-07-2	ACID3	272.72 ^ab^	12	250.15 ^ab^	20	54.32 ^c^	8		325.28 ^a^	7	255.01 ^ab^	17	91.84 ^c^	10		200.62 ^b^	6	195.79 ^b^	14	41.50 ^c^	8	0.2	<0.0001 *
24	Decanoic acid	334-48-5	ACID4	18.73 ^b^	4	20.77 ^b^	25	4.68 ^d^	8		30.21^ab^	6	8.57 ^cd^	26	6.42 ^cd^	3		21.22 ^ab^	2	15.37^bc^	2	4.22 ^d^	4	0.9	0.0004 *
25	Benzoic acid	65-85-0	ACID5	65,564 ^ab^	8	59,455 ^abcd^	13	53162 ^bcd^	10		60,490^abcd^	7	47,267 ^d^	10	49,584 ^cd^	4		72,760 ^a^	3	59,848^abcd^	3	64,506 ^abc^	9	0.0003 *	0.0019 *
***Others***																									
26	Pentanal	110-62-3	PEN	11.55 ^a^	2	13.65 ^a^	5	6.76 ^a^	8		9.41^a^	5	15.47 ^a^	10	6.43 ^a^	14		6.76 ^a^	8	7.80^ab^	7	7.06 ^ab^	7	0.9	0.1
27	3-Octanone	106-68-3	OCT	0.82 ^bc^	24	0.67 ^bcd^	16	0.16 ^e^	15		1.21^a^	13	0.92 ^ab^	17	0.90 ^ab^	13		0.49 ^cde^	2	0.58^bc^	6	0.30 ^c^	16	<0.0001 *	0.0032 *
28	D-Limonene	5989-27-5	LIM	7.47 ^cd^	2	6.45 ^e^	6	6.51 ^de^	1		6.87^cde^	2	6.42 ^e^	7	7.66 ^bc^	2		8.72 ^ab^	4	9.36^a^	8	8.97 ^a^	2	<0.0001 *	0.2
29	β-damascenone	23696-85-7	DAM	1.40 ^b^	8	0.93 ^b^	11	0.75 ^b^	30		1.32^b^	9	0.95 ^b^	35	4.96 ^a^	15		1.18 ^b^	4	1.23^b^	19	0.75 ^b^	24	0.0494 *	0.3
30	4-ethylguaiacol	2785-89-9	EG	8.46 ^a^	10	8.35 ^a^	16	6.79 ^ab^	4		5.67 ^b^	6	0.52 ^d^	16	1.47 ^cd^	9		3.07 ^bc^	3	2.23 ^c^	2	1.56 ^d^	2	<0.0001 *	0.0042 *
31	4-ethylphenol	123-07-9	EP	20.31 ^a^	12	11.32 ^b^	10	11.82 ^b^	4		6.25 ^c^	2	2.49 ^d^	3	4.05 ^cd^	3		3.16 ^cd^	1	3.15 ^c^	1	3.88 ^cd^	1	<0.0001 *	0.0012 *

Values identified by the same letters are not significantly different at the 0.05 level (Tukey-Kramer HSD test). *: significant impact on the compound.

**Table 4:** OAVs (odour activity values) and descriptors of volatile compounds detected in cranberry wines.

No.	Compounds	Descriptor ^a^	OAV											Thresholds (µg/kg) ^b^
			Stevens				Pilgrim				Bergman			
			White	Thermo	Red		White	Thermo	Red		White	Thermo	Red	
1	Ethyl Acetate	floral	1143	962	1130		1143	986	1381		1116	821	1269	5
2	Methyl butyrate	apple	<1	<1	<1		2	1	2		1	1	1	1
3	Ethyl butyrate	fruity	358	336	390		450	469	531		390	304	345	0.1
4	Ethyl methylbutyrate	fruity	1109	911	1165		1071	1153	1324		1127	785	943	0.01
5	Isoamyl acetate	banana	14	13	19		16	16	29		15	13	20	2
6	Ethyl hexanoate	fruity	120	99	55		141	126	95		76	75	42	0.3
7	Methyl benzoate	herb	<1	<1	<1		<1	<1	1		<1	<1	1	110
8	Ethyl octanoate	fruity	9	7	1		10	8	3		5	6	1	5
9	Ethyl benzoate	floral, fruity	38	27	16		49	17	31		41	31	24	100
10	Ethyl cinnamate (*trans*)	floral	5	6	1		10	5	2		4	3	2	17
11	Isobutanol	wine	33	33	38		44	42	46		31	21	43	360
12	Isoamylalcohol	whiskey, pungent	105	103	107		116	108	120		101	101	96	250
13	α-terpineol	fresh, minty	10	8	10		10	10	11		15	15	17	28
14	Benzyl alcohol	floral	1535	1473	2170		765	627	1536		985	1273	1487	1.2
15	Geraniol (*trans*)	rose	17	15	16		21	21	24		19	16	18	4
16	Phenylethyl alcohol	green, floral	207,752	193,581	190,631		273,855	220,509	251,881		199,320	185,010	183,453	0.015
17	Methylbutyric acid	cheese, sweat	52	43	29		54	46	32		50	37	33	10
18	Hexanoic acid	sweat	3	5	3		5	6	4		3	3	2	93
19	Pentanal	pungent	<1	1	<1		<1	1	<1		1	<1	<1	12
20	D-Limonene	citrus, mint	2	2	2		2	2	2		2	2	2	4
21	β-damascenone	apple, rose, honey	1993	1328	1069		1888	1353	7085		1683	1763	1074	0.0007

^a^, ^b^: The descriptor and threshold were found in the literature [27].

## Supplementary Materials

The following are available online at https://www.mdpi.com/2076-3921/8/5/144/s1, Figure S1: Flowchart of cranberry wine fermentation, Table S1: List of multiple reaction monitoring (MRM) parameters for identification of phenolic acids, flavonols, and proanthocyanins in cranberry wines.
