# Supplementary material for: The Influence of Vinification Methods and Cultivars on the Volatile and Phenolic Profiles of Fermented Alcoholic Beverages from Cranberry
Source: Antioxidants (Basel). 2019 May 23;8(5):144. doi: 10.3390/antiox8050144 (PMC6562399; doi:10.3390/antiox8050144)
Supplement: Supplementary file 1 [file antioxidants-08-00144-s001.pdf]

Supplementary Materials

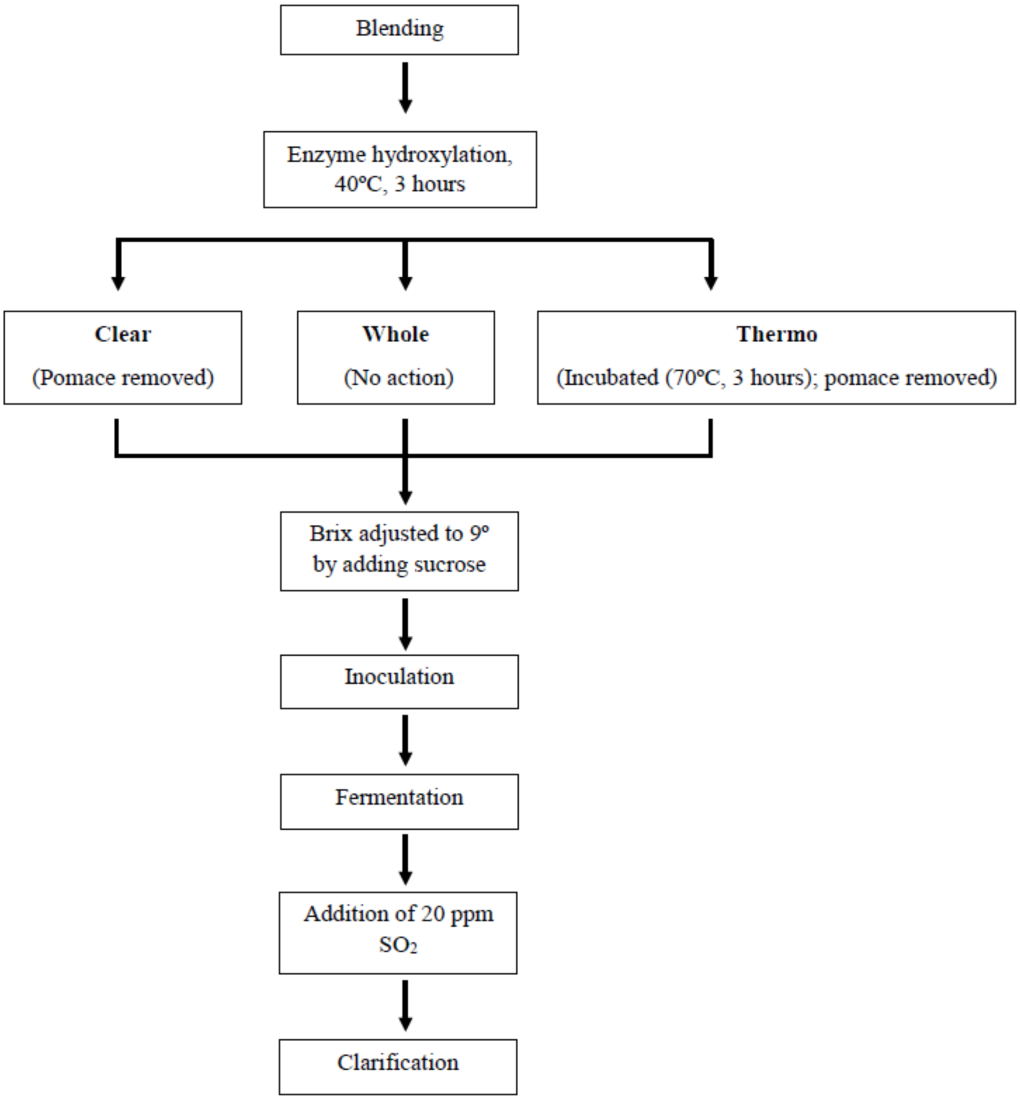

Figure S1. Flowchart of cranberry wine fermentation.

Table S1. List of Multiple Reaction Monitoring (MRM) parameters for identification of phenolic acids, flavonols, and proanthocyanins in cranberry wines.

No.	Compounds	Retention Time (min)	*m/z* Precursor ion (Th)	*m/z* Quantifier ion (Th)	Energy (V)	*m/z* Qualifier ion (Th)	Energy (V)
1	Gallic acid	1.28	169	125	10	79	25
2	Protocatechuic acid	2.16	153	109	10	108	25
3	Procyanidin B1	3.51	577	289	15	245	15
4	*m*-Salicylic acid	3.72	137	93	10	137	0
5	Gentisic acid	4.59	153	109	10	108	25
6	Catechin	4.68	289	245	10	203	15
7	β-Resorcylic acid	5.09	153	109	10	65	15
8	Chlorogenic acid	5.21	353	191	15	85	40
9	Vanillic acid	5.44	167	152	10	167	0

10	*p*-Salicylic acid	5.57	137	93	10	137	0
11	Caffeic acid	5.71	179	135	10	89	40
12	Procyanidin B2	6.30	577	289	15	407	15
13	Epicatechin	7.61	289	245	10	109	25
14	*p*-Coumaric acid	8.45	163	119	10	163	0
15	Myricetin-3-galactoside	9.86	479	316	20	271	40
16	Myricetin-3-glucoside	10.09	479	316	20	271	40
17	*trans*-Sinapic acid	10.59	223	208	10	193	25
18	Quercetin-3-galactoside	11.39	463	300	18	270	40
19	Procyanidin A2	11.52	575	285	10	243	25
20	Quercetin-3-rhamnoside	12.90	447	301	10	300	25
21	Phloridzin	14.76	435	167	30	123	40
22	Quercetin	18.68	301	151	20	179	10
23	Kaempferol	23.33	285	93	22	285	0
